# The Promoting Mechanism of the Sterile Fermentation Filtrate of *Serratia odorifera* on *Hypsizygus marmoreus* by Means of Metabolomics Analysis

**DOI:** 10.3390/biom13121804

**Published:** 2023-12-18

**Authors:** Jixuan Cao, Jiacheng Xie, Mingming Yu, Tao Xu, Huangru Zhang, Liding Chen, Shujing Sun

**Affiliations:** 1 College of Life Sciences, Fujian Agriculture and Forestry University, Fuzhou 350002, China; 2210514023@fafu.edu.cn (J.C.); 3200537080@fafu.edu.cn (J.X.); 1210514112@fafu.edu.cn (M.Y.); 3215413137@stu.fafu.edu.cn (T.X.); 3205403014@stu.fafu.edu.cn (H.Z.); chenliding@fafu.edu.cn (L.C.); 2 Gutian Edible Fungi Research Institute, Fujian Agriculture and Forestry University, Ningde 352200, China

**Keywords:** *H. marmoreus*, *S. odorifera*, promoting mechanism, metabolomics analysis

## Abstract

*Hypsizygus marmoreus* has become one of the most popular edible mushrooms due to its high nutritional and economic value. Previous researchers found that *Serratia odorifera* could promote the growth of *H. marmoreus* by producing and secreting some of its inducers. However, the specific mechanism of action was still unclear. In this study, we found that the exogenous addition of sterile fermentation filtrate (HZSO-1), quorum sensing (QS) signaling molecules, 3-oxo-C_6_-HSL, cyclo(Pro-Leu), and cyclo(Tyr-Leu) could significantly promote the growth of *H. marmoreus*, increase the number of clamp junctions, and the diameter of mycelium (*p* < 0.05). In addition, non-targeted metabolomic analysis revealed that 706 metabolites were detected in the treated group. Of these, 307 metabolites were significantly different (*p* < 0.05). Compared with the control, 54 and 86 metabolites were significantly increased and decreased in the HZSO-1 group, respectively (*p* < 0.05). We speculate that the sterile fermentation filtrate of *S. odorifera* could mediate the carbohydrate and amino acid metabolism of *H. marmoreus* by influencing the pentose phosphate pathway (PPP) to increase the energy supply for the growth and development of the mycelium. The above results will further reveal the growth-promoting mechanism of *S. odorifera* on *H. marmoreus*.

## 1. Introduction

*Hypsizygus marmoreus* is a commercial edible mushroom with a high nutritional value, and it is widely cultivated in East Asia. It has become one of the most popular edible mushrooms due to its high nutritional value and medicinal value [1]. However, there are many problems in its production, such as high planting costs, long growth cycles, and low economic benefits. Therefore, it is of great importance to find new ways to promote the development of the industrial chain of *H. marmoreus* by shortening the production cycle, increasing the yield, and promoting the quality and safety of agricultural products. The use of growth-promoting microorganisms, as one of the most promising biological approaches for the improvement of crop growth and yield, has been widely applied in ecological and agriculturally friendly cultivation worldwide [2]. A large number of studies have shown that the population of potent microorganisms plays an important role in the growth of mycelium and other stages in the cultivation of mushrooms [3,4]. Kertesz et al. recently reviewed a number of bacterial and fungal strains, including *Bacillus*, *Bradyrhizobium*, and *Pseudomonas*, indicating that they could promote fungal growth [4]. Cho et al. showed that fluorescent *Pseudomonas* spp. isolated from mushroom mycelia could promote the formation of primordium and the development of the basidiophore of *Pleurotus ostreatus* [5]. Similarly, in the previous study, the predominant symbiotic bacteria (*Serratia* genera, HZSO-1) was isolated from *H. marmoreus* cultivation bags, which could promote the growth and development of *H. marmoreus*, shorten the growth cycle, and improve the yield of the fruiting body [6].

*Serratia* species are Gram-negative γ-proteobacteria with ubiquitous distribution. Currently, *Serratia* species are studied for their potential to act as antifungal agents [7]. As an effective biological control factor, it could resist plant soil and leaf diseases. It has also been considered an effective factor for entomopathogenic microorganisms and weed suppression [8,9]. However, there are few reports on the interaction between *Serratia* species and fungal growth. During the growth process, the bacteria would synthesize and secrete some auto-induced, which could release small molecular chemical signal molecules, which enter and exit the cell membrane freely into the surrounding environment [10]. With the increase in cell density, foreign signal molecules could continue to accumulate until reaching the critical concentration, inducing or inhibiting the expression of a series of genes, thus regulating the growth of bacteria [11]. A considerable amount of existing research has shown that bacteria could regulate many important biological functions through the exchange of quorum-sensing molecules (QSMs) [11,12,13]. N-acyl homoserine lactones (AHLs) and modified oligopeptides, as typical representatives of QSMs, are widely distributed in microorganisms, which could have a strong impact on the physiological metabolism of microorganisms [14]. Von Rad et al. showed that the quorum sensing signal C_6_-HSL produced by bacteria could promote the growth of *Arabidopsis thaliana* roots and induce the alteration of growth hormones in plants [15]. Ortíz-Castro et al. have shown that AHL signal molecules with different carbon lengths could stimulate the growth of *A. thaliana* roots [16]. In particular, C_10_-HSL could affect primary root growth, lateral root formation, and root hair development. At present, many kinds of AHL signal molecules have been reported in *Serratia*, which play different roles in the interaction with plants [17,18,19]. However, there have been few studies on the effects of QSMs produced by *Serratia* on the growth and development of edible mushrooms. In our previous research, we discussed the effect of quorum sensing mediated by signaling molecules such as cyclic dipeptides and N-acyl homoserine lactones produced by *S. odorifera* on the activities of extracellular laccase and manganese peroxidase of *H. marmoreus* [20]. However, the molecular mechanism by which the symbiotic *S. odorifera* HZSO-1 promotes the growth and development of *H. marmoreus* mycelium and its effect on the metabolic pathway and metabolites of the mycelium will be further investigated.

Therefore, based on the previous work, this research has investigated the effect of signaling molecules of *S. odorifera* on the growth and development of *H. marmoreus* mycelium by adding the sterile fermentation filtrate and QS signaling molecules of *S. odorifera*. Moreover, metabolomic analysis was applied to further reveal the interaction mechanism between *S. odorifera* and *H. marmoreus* mycelium, deepening the understanding of the bacterial–fungal interaction.

## 2. Materials and Methods

### 2.1. Strain Culture and Chemical Synthesis

The *H. marmoreus* and *S. odorifera* strains used in the experiment were obtained from the strains maintained by our team (Special Edible Mushroom Species Innovation and Metabolic Engineering Team of Fujian Agriculture and Forestry University, Fuzhou, China). *H. marmoreus* was grown at 25 °C in PDA liquid medium (2% glucose, 0.3% yeast extract, 0.3% tryptone, 0.15% KH_2_PO_4_, 0.15% MgSO_4_-7H_2_O, and 0.01% vitamin B1 (VB1) in 1 L of potato infusion). *S. odorifera* was incubated in LB liquid medium (1 L LB liquid medium containing peptone 10 g, yeast 5 g, NaCl 10 g, agar 20 g, pH 7.2–7.4) at 30 °C with shaking at 180 rpm.

The signal molecules were synthesized by chemical synthesis from Sangon Biotech Company (Shanghai, China). Other chemicals used in this study were purchased from Sinopharm Chemical Reagent Co. (Shanghai, China). All the samples of the signal molecules were dissolved in 5% acetonitrile, filtered through an organic filter membrane (0.22 μm), and stored at −20 °C.

### 2.2. Screening of the Optimal Concentration of the Sterile Broth of S. odorifera

An aseptic fermentation broth of HZSO-1 was prepared according to the method of [6]. Plates were prepared with PDA medium containing different amounts of sterile fermentation filtrate (3% *v*/*v*, 5% *v*/*v*, 7% *v*/*v*). PDA medium without fermentation filtrate was used as a control. A cake (Ø = 9 mm) of *H. marmoreus* mycelium was placed in the middle plate and incubated at 25 °C. The diameter of the mycelium was measured every 4 d using an electronic vernier caliper, and the growth rate was calculated. Each treatment was replicated three times, and each replicate consisted of three plates. The optimal volume of HZSO-1 sterile fermentation broth was selected and used for the subsequent experiments.

### 2.3. Screening for Optimal Concentration of Signal Molecule Markers

The signal molecule standard mother solution was obtained by 2.1; acetonitrile of the same concentration was added as a control. The PDA plate was prepared with three concentration gradients of 5 μg/mL, 10 μg/mL, and 20 μg/mL, respectively. After inoculation with *H. marmoreus*, the optimum concentration of the single signal molecular standard was screened by calculation of its growth rate. On this basis, the optimal concentrations of 3-oxo-C_6_-HSL-10 μg/mL, cyclo(Pro-Leu)-20 μg/mL, and cyclo(Tyr-Leu)-10 μg/mL were determined. They were used as references for subsequent screening experiments to determine the concentration of mixed signaling samples. The mixed groups (3-oxo-C_6_-HSL: cyclo(Pro-Leu):cyclo(Tyr-Leu) in 1:1:1 ratio) were tested with 2 μg/mL, 5 μg/mL, and 10 μg/mL of each sample. After inoculation with *H. marmoreus* mycelium, the optimal concentration of the mixed signal molecule was selected by calculating its growth rate.

### 2.4. Mycelium Ultrastructure Observation

The optimal fermentation filtrate and the optimal concentrations of the single signal molecule and the mixed standard were investigated in four treatments, 3-oxo-C_6_-HSL-10 μg/mL, cyclo(Pro-Leu)-20 μg/mL, and cyclo(Tyr-Leu)-10 μg/mL, and mixed-5 μg/mL. The same concentration of acetonitrile was added to HZSO-1 and the control to reduce the error. After inoculation with *H. marmoreus* mycelia, plates were incubated at 25 °C for 14 d, and sections were prepared according to [21]. The ultrastructure of *H. marmoreus* was observed using scanning electron microscopy (SEM, ZEISS ULTRA 55 Oberkochen, Germany).

### 2.5. Metabolite Extraction and Machine Detection Conditions

First, 25 mg of mycelial samples were accurately weighed and added to 500 µL of extract (methanol:acetonitrile:water = 2:2:1 (*v*/*v*), internal standard mixture with isotopic labels). The samples then underwent milling treatment at 35 Hz for 4 min, ultrasound treatment in an ice bath for 5 min, repeated 3 times; treated samples were stored at −40 °C for 1 h; centrifugation at 12,000 rpm at 4 °C for 15 min; the supernatant was collected in a sample bottle for machine analysis. All samples were then mixed with equal supernatant into machine test QC samples [22].

A Vanquish (Thermo Fisher Scientific, Waltham, MA, USA) ultra-high performance liquid chromatograph was used in the study. The target compounds were separated on a Waters ACQUITY UPLC BEH Amide (2.1 mm × 100 mm, 1.7 µm) liquid chromatography column. The liquid chromatography consisted of an aqueous phase A, 25 mmol/L ammonium acetate and 25 mmol/L ammonia water, and an acetonitrile phase B. The automated injection temperature was 4 °C, and the sample size was 2 μL.

The Orbitrap Exploris 120 mass spectrometer was controlled by the control software (Xcalibur, version 4.4, Thermo) for primary and secondary mass spectrum data collection. Detailed parameters were as follows. Sheath gas flow, 50 Arb; auxiliary gas flow, 15 Arb; capillary temperature, 320 °C; full MS resolution, 60,000; MS/MS resolution, 15,000; collision energy in NCE mode, 10/30/60; spray voltage, 3.8 kV (positive ion mode) or −3.4 kV (negative ion mode).

### 2.6. UHPLC-QE-MS Analysis

#### 2.6.1. Preprocessing of Original Data and Metabolite Description

In order to reduce the effect of the detection system error on the results and to better highlight the biological significance of the results, the original data were preprocessed. It mainly included the following steps: deviation filtering; missing value filtering; missing value filling; data standardization processing. After a series of preprocessing of the original data, all metabolites were searched and collected in the local database for material information, including common database (HMDB, KEGG COMPOUND) numbering index, Chinese information, classification information, etc. After the original data were converted into mzXML format using ProteoWizard (3.0.20233) software, the independent R program package (kernel is XCMS) was used for peak identification, peak extraction, peak alignment, and integration, and then material annotation was performed through comparison with the BiotreeDB (V2.1) self-built secondary mass spectra database.

#### 2.6.2. Metabolite Classification Statistics

The identified metabolites were classified and counted according to the information of chemical classification. The classification of metabolites and the proportion of different substances were obtained.

#### 2.6.3. Multivariate Statistical Analysis

SIMCA (v16.0.2, Sartorius Stedim Data Analytics AB, Umea, Sweden) software was used for principal component analysis (PCA), partial least squares discriminant analysis (PLS-DA), and orthogonal partial least squares discriminant analysis (OPLS-DA). Meanwhile, to verify the quality of the OPLS-DA model, 7-fold cross-validation was used. Then, R2Y (the model’s interpretability to classification variable Y) and Q (the model’s predictability) obtained after cross-validation were used to evaluate the model’s effectiveness. Finally, the permutation test was used to obtain different random Q values by randomly changing the order of Y several times. The validity of the model was further tested.

#### 2.6.4. Screening and Analyzing Differential Accumulated Metabolites (DAMs)

On the basis of *p* < 0.05 of the *t*-test in univariate statistical analysis and the threshold condition of VIP > 1 of variable predicted importance of the first principal component of the OPLS-DA model in multivariate statistical analysis, the DAMs between five treatment groups and the control group were screened. DAMs between the five treatment groups and control group were screened with threshold conditions of *p* < 0.05 and VIP > 1 of the first principal component variable of the OPLS-DA model. Volcano maps were drawn to visualize the overall metabolite distribution between groups. After screening the DAMs, the Euclidean distance matrix was calculated for the quantitative values of each group of DAMs. The DAMs were clustered using the complete linkage method, which was displayed in the form of a heat map. Then, the number of DAMs in each comparison group was illustrated with Venn diagrams.

#### 2.6.5. Metabolic Pathway Analysis of DAMs

The metabolic pathways of DAMs obtained in 2.6.4 were analyzed using the Kyoto Encyclopedia of Genes and Genomes (KEGG) database. Their metabolic pathways were jointly analyzed, and KEGG pathway enrichment analysis was performed. The metabolic response mechanism of *H. marmoreus* mycelium to QS signaling molecules was determined using metabolomics. Metabolomics analysis included six replicates per treatment.

### 2.7. Statistical Analysis

The data were analyzed with analysis of variance (ANOVA) using SPSS software (version 17.0). Comparisons between means were determined by Duncan’s multiple range test (*p* < 0.05).

## 3. Results

### 3.1. Effect of Sterile Fermentation Broth of S. odorifera and Different QS-Signaling Molecules on Mycelial Growth of H. marmoreus

As shown in Figure S1, mycelial growth was significantly faster (*p* < 0.05) with added sterile fermentation broth compared to the control, and the fastest growth was achieved at 5% added broth. Meanwhile, the mycelium was denser at 5% addition. Therefore, in subsequent experiments, the optimal 5% addition of sterile fermentation broth was selected.

Based on the solubilization properties of different standards, cyclo(Pro-Leu), cyclo(Tyr-Leu), 3-oxo-C_6_-HSL, and cyclo(Tyr-Ile) were solubilized by adding acetonitrile (5%), and the same acetonitrile concentration was used as control. It was found that cyclo(Pro-Leu), cyclo(Tyr-Leu), and 3-oxo-C_6_-HSL could promote *H. marmoreus* mycelial growth, and their optimal concentrations were 20 μg/mL, 10 μg/mL, and 10 μg/mL, respectively (Figure S2). However, there was no significant difference in the growth-promoting effect of cyclo(Tyr-Ile) among the three concentration gradients (*p* > 0.05). Cyclo(Pro-Phe) standard products need to be dissolved with formic acid, and it was found that adding formic acid severely inhibited mycelial growth (Figure S2). Therefore, the optimal concentrations of cyclo(Pro-Leu), cyclo(Tyr-Leu), and 3-oxo-C_6_-HSL were used in subsequent experiments to determine whether the mixture of signaling molecules would affect the growth of *H. marmoreus*. The three signaling molecules, cyclo(Pro-Leu), cyclo(Tyr-Leu), and 3-oxo-C_6_-HSL, were mixed according to their optimal concentrations, and a 1:1:1 ratio of 2 μg/mL, 5 μg/mL, and 10 μg/mL of each was set for the assay. As shown in Figure S3, there was no significant difference in growth rate with the exogenous addition of the mixed standard of 5 μg/mL and 10 μg/mL in comparison with the control, but the mycelium grew more densely.

**Figure 1:**
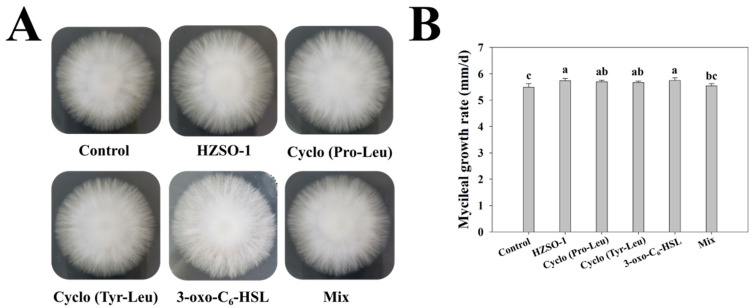
(**A**) Diagram and (**B**) mycelial growth rate (mm/d) of *H. marmoreus* after treatment with sterile fermentation filtrate of *S. odorifera* and different QS signaling molecules.

The addition of both the aseptic fermentation filtrate of *S. odorifera* and the signal molecule marker promoted the growth of *H. marmoreus* mycelium. However, the mycelium grew faster after HZSO-1 and 3-oxo-C_6_-HSL treatments, and the mycelium was denser in the mixed treatment group (Figure 1). Therefore, the treatment group, HZSO-1 (5%), cyclo(Pro-Leu)-20 μg/mL, cyclo(Tyr-Leu)-10 μg/mL, 3-oxo-C_6_-HSL-10 μg/mL, mix-5 μg/mL and their control were selected for metabolomic analysis in the following experiment to further explore the mechanism of the interaction between the signaling molecules in the sterile fermentation filtrate of *S. odorifera* and the mycelium of *H. marmoreus*.

### 3.2. Effect of Sterile Fermentation Filtrate of S. odorifera and Different QS Signal Molecules on the Microstructure of H. marmoreus Mycelium

**Figure 2:**
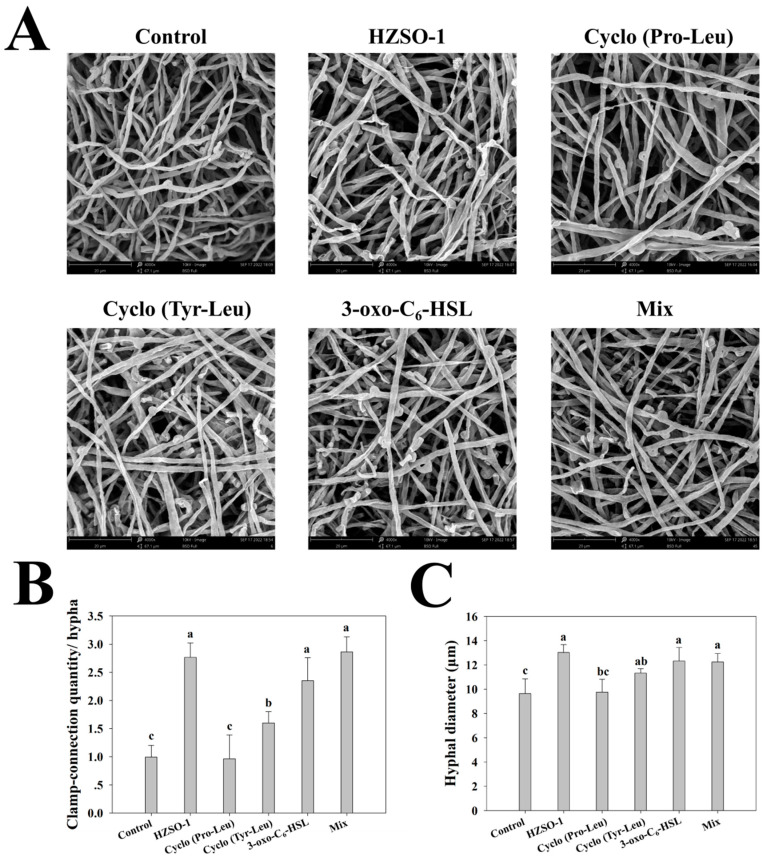
(**A**) Microscopic structure by SEM; (**B**) number of clamp junctions per hypha and (**C**) hypha diameter (μm) of *H. marmoreus* mycelium after treatment with sterile fermentation filtrate of *S. odorifera* and different QS signal molecules. Means with different letters are significantly different at *p* < 0.05.

To further investigate the effect of signaling molecules in the sterile fermentation filtrate of *S. odorifera* on the mycelial growth of *H. marmoreus*, scanning electron microscopy was performed on the plate covered with mycelium. The results showed that the number of clamp junctions in the three treatment groups, HZSO-1, 3-oxo-C_6_-HSL, and mix, was significantly higher than that in the control group (*p* > 0.05). The number of clamp junctions in the HZSO-1 treatment group was approximately 2.79 times higher than that in the control group (Figure 2A,B). The mycelium was more robust after treatment with HZSO-1 and signaling molecular probe; the mycelium diameter was 3.40 higher in the HZSO-1-treated group and 2.61 higher in the mix-treated group than in the control group (Figure 2C).

### 3.3. Effect of Sterile Fermentation Filtrate of S. odorifera and Different QS Signal Molecules on the Metabolome of H. marmoreus Mycelium

**Figure 3:**
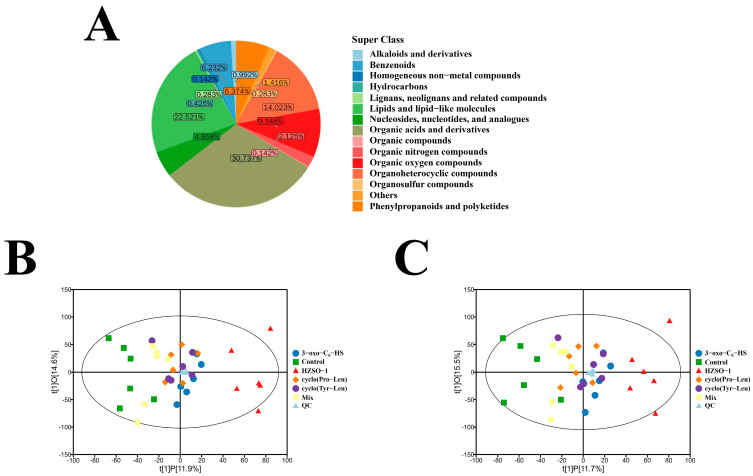
Overview of metabolomics in *H. marmoreus* mycelium. (**A**) Pie chart of the metabolite classification and the proportion of different substances; (**B**) OPLS-DA plot of *H. marmoreus* mycelium from six experimental groups in the positive ion mode; (**C**) in the negative ion mode.

Total ion chromatography (TIC) of QC samples from six groups of *H. marmoreus* mycelium was performed, as shown in Figure S4. In the original data of positive and negative ion mode, 12,487 and 12,722 peaks were extracted, respectively, and 10,671 and 10,682 peaks were retained after pretreatment. After qualitative matching analysis using secondary mass spectrometry, a total of 706 metabolites were identified. Of these, 503 metabolites were found in the positive ion mode, and 250 metabolites were found in the negative ion mode (47 metabolites were common to both modes). As shown in Figure 3A, these metabolites were divided into 15 groups at the superclass level, mainly including organic acids and derivatives (30.74%), lipids and lipid-like molecules (22.52%), organic heterocyclic compounds (14.02%), organic oxygen compounds (9.35%), and phenylpropanoid and polyketide compounds (6.37%).

Principal component analysis (PCA), partial least squares discriminant analysis (PLS-DA), and orthogonal partial least squares discriminant analysis (OPLS-DA) were used to distinguish the accumulation of metabolites in *H. marmoreus* mycelium. PCA analysis of the metabolites of the QC samples showed that the QC samples were clustered and close to the origin, which was an indication that the experimental results were stable and reliable. *H. marmoreus* mycelium distribution under six different treatments in PCA plots showed that most samples were distributed within a 95% confidence interval, but separation between groups was not obvious. In addition, the contribution rates of the first principal component (PC1) and the second principal component (PC2) under the positive and negative ion models were 16.80% and 17.90%, respectively. The cumulative contribution rates of the x-axis model were 0.523 and 0.522, respectively. Therefore, the PCA model had a high degree of fit (Figure S5A,B). Based on the above PCA analysis, we further analyzed metabolite data using OPLS-DA models, extracting metabolic difference information between treatment groups. It could be seen from the OPLS-DA models that HZSO-1, 3-oxo-C_6_-HSL, cyclo(Pro-Leu), cyclo(Tyr-Leu), and mix were clearly separated from control, indicating that the metabolic profiles of *H. marmoreus* mycelium had different changes after the addition of QS signaling molecules. The metabolic profiles of *H. marmoreus* mycelium under each treatment were also different (Figure 3B,C). Regarding HZSO-1 and control, there was some overlap in the PCA plots (Figure S5A,B), but clear distinctions were observed in the OPLS-DA models, indicating that the metabolism of *H. marmoreus* mycelium was altered after HZSO-1 treatment. These results indicated that the information on differential metabolites between *H. marmoreus* mycelium treated with different QS signaling molecules and the control group was reliable. DAMs could be screened according to the variable importance projection (VIP) value.

### 3.4. Screening and Analysis of the DAMs

**Figure 4:**
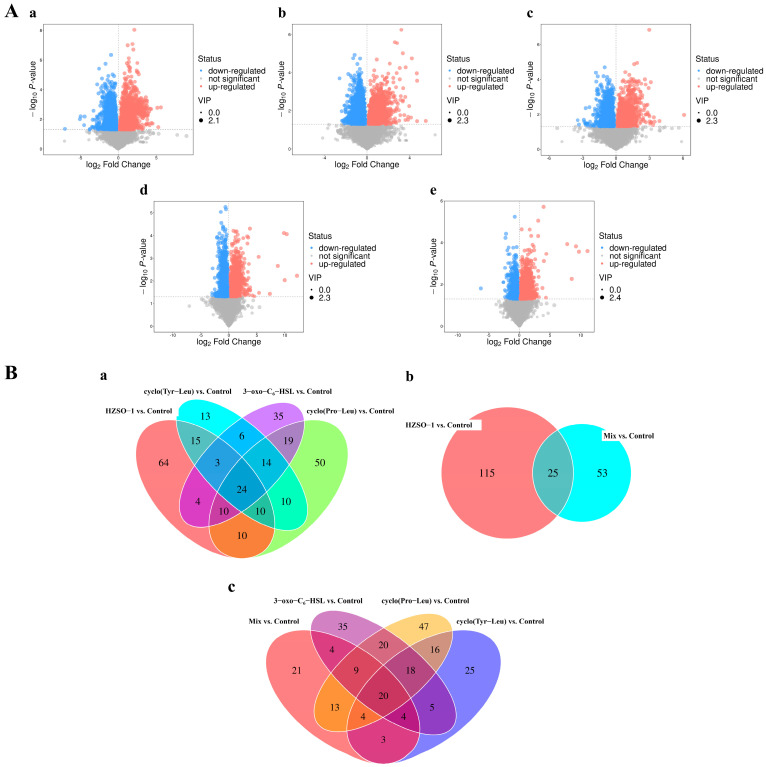
(**A**) Differential accumulation of metabolites screening volcano map of *H. marmoreus* mycelia samples in five groups. (**a**) HZSO-1 vs. control; (**b**) cyclo(Pro-Leu) vs. control; (**c**) cyclo(Tyr-Leu) vs. control; (**d**) 3-oxo-C_6_-HSL vs. control; (**e**) mix vs. control. (Red—upregulated DAMs; Blue—downregulated DAMs; Gray—not significant DAMs). (**B**) Venn diagram of differential accumulation metabolites in different comparison groups. (**a**) cyclo(Pro-Leu) vs. control, cyclo(Tyr-Leu) vs. control, 3-oxo-C_6_-HSL vs. control, and HZSO-1 vs. control; (**b**) mix vs. control, and HZSO-1 vs. control; (**c**) mix vs. control, cyclo(Pro-Leu) vs. control, cyclo(Tyr-Leu) vs. control, and 3-oxo-C_6_-HSL vs. control.

The VIP of the OPLS-DA model was combined with the *p*-value of the univariate statistical analysis. VIP > 1 and *p* < 0.05 were taken as the thresholds to screen DAMs, and a DAMs screening volcano diagram among all groups was obtained (Figure 4A). Based on the qualitative matching results of secondary mass spectrometry, the final DAMs were obtained, and the differential substances in *H. marmoreus* mycelium of each group were analyzed using a cluster heat map. A total of 140 DAMs were identified in the metabolites of *H. marmoreus* mycelium in the HZSO-1 vs. control group, accounting for 19.83% of all secondary identified metabolites, as shown in Figure S6. Among the 140 DAMs, the relative contents of 54 components in the HZSO-1 group were significantly higher than those in the control group (*p* < 0.05). α-L-glutamyl-L-glutamic acid was the most upregulated compound (fold change = 4.74), the relative contents of 86 components were significantly lower than those in the control group, and ADP was the most downregulated compound (fold change = 0.14). A total of 147 DAMs were identified in the sampled metabolites of cyclo(Pro-Leu) vs. the control group, accounting for 20.82% of all secondary metabolites. A total of 95 DAMs were identified in the metabolites of the *H. marmoreus* mycelium in the cyclo(Tyr-Leu) vs. control group. A total of 115 DAMs were identified in the 3-oxo-C_6_-HSL versus control. The number of DAMs identified in the mix vs. control group was the lowest, with a total of 78 DAMs representing 11.05% of all identified metabolites, of which 54 DAMs were upregulated in the mix group compared to the control. The 13, 14-Dihydro-15-Keto-pgd2 (fold change = 4.50) was the most upregulated, and 24 types of fatty acids were downregulated, of which ginkgetin (fold change = 0.46) was the first to be downregulated.

As shown in Figure 4(Ba), the number of unique DAMs in *H. marmoreus* mycelium under the addition of HZSO-1 was the largest compared to other QS signal molecules, with a total of 64, followed by the cyclo(Pro-Leu) group with a total of 50. From the perspective of the intersection of HZSO-1 with other groups, HZSO-1, cyclo(Pro-Leu), cyclo(Tyr-Leu), and 3-oxo-C_6_-HSL shared 54, 52, and 41 DAMs, respectively. As shown in Figure 4(Bb), only 25 DAMs were shared between HZSO-1 and mix, while 115 unique DAMs were found in the HZSO-1 group. Moreover, 46 DAMs were shared between cyclo(Pro-Leu) and mix group, 31 DAMs were shared between cyclo(Tyr-Leu) and mix, and 37 DAMs were shared between 3-oxo-C_6_-HSL and mix (Figure 4(Bc)).

The above results show that the addition of HZSO-1 and cyclo(Pro-Leu) had a great effect on the metabolic activity of *H. marmoreus* mycelium, and the mix addition of [3-oxo-C_6_-HSL + cyclo(Pro-Leu) + cyclo(Tyr-Leu)] −5 μg/mL had the least metabolic disturbance on *H. marmoreus* mycelium. The effect of HZSO-1 on *H. marmoreus* mycelial metabolites could not be replaced by the other three single or mixed QS signaling molecules.

### 3.5. Analysis of the Pathways of the Different Metabolites

After the DAMs between the five treatment groups and the control group were matched with the KEGG database, the 15 KEGG pathways with the highest concentration of DAMs in each group were shown using a classification map, and then the pathways with statistical significance with *p* < 0.05 as the threshold were screened.

**Figure 5:**
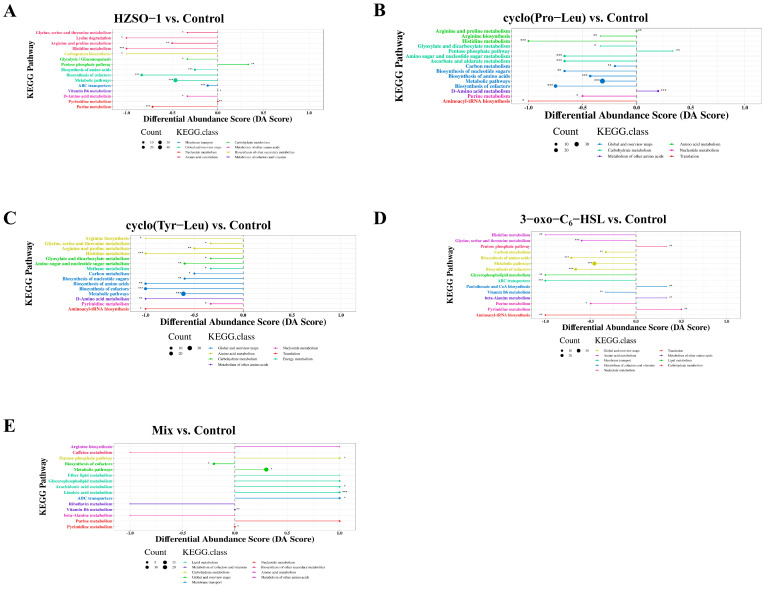
KEGG classification for different comparison groups. (**A**) HZSO-1 vs. control; (**B**) cycle (Pro-Leu) vs. control; (**C**) cycle (Tyr-Leu) vs. control; (**D**) 3-oxo-C_6_-HSL vs. control; (**E**) mix vs. control. Different letters mean a significant difference (* 0.01 < *p* < 0.05, ** 0.001 < *p* < 0.01, *** *p* < 0.001).

As shown in Figure 5, the addition of sterile fermentation filtrate of *S. odorifera* and QS signaling molecules had the following effects on the metabolic pathway of *H. marmoreus* mycelium: amino acid metabolism, membrane transport, lipid metabolism, cofactor and vitamin metabolism, nucleotide metabolism, translation, biosynthesis of other secondary metabolites, energy metabolism, and carbohydrate metabolism. The pentose phosphate pathway (PPP) in the carbohydrate metabolism pathway was significantly upregulated after HZSO-1 treatment and was also found to be significantly upregulated in the cyclo(Pro-Leu), 3-oxo-C_6_-HSL, and mix treatment groups. In each treatment group, amino acid metabolism was also enriched. Among them, arginine and proline metabolism were significantly downregulated in HZSO-1, cyclo(Pro-Leu), and cyclo(Tyr-Leu) groups.

## 4. Discussion

In recent years, with the rapid development of microbiomics and cultureomics-related technologies, technical means have been provided to obtain highly active functional strains with promotive effects. Beneficial microorganisms not only enhance the growth and yield of edible mushrooms but also increase the resistance of the cultivated strains to harmful microorganisms and, thus, shorten the growth cycle [2,23]. Two *Pseuodomonas putida* strains (Bt4 and Ps7) isolated from mushroom farms could increase *Agaricus bisporus* production and be used as potential growth-promoting inoculants for production [24]. Kumari et al. showed that MRC119 improved oyster mushroom growth and yield [2]. In addition, in a previous study, the application of *Bacillus subtilis* at a lower concentration promoted mushroom yield [23]. Therefore, the role of beneficial microorganisms in different growth and development stages of edible mushrooms has become a research hotspot in recent years. *H. marmoreus*, an important edible and medicinal fungus, is one of the main species of edible mushrooms for factory cultivation in China [1]. In the previous experiment, high-throughput sequencing technology was used to analyze the culture packages of *H. marmoreus*, and it was found that there was a better growth-promoting effect of *S. odorifera* on *H. marmoreus* [6]. However, the exact mechanism of action is not clear. In this experiment, metabolomic analysis was performed to systematically elucidate the growth-promoting mechanism. Furthermore, the interaction between edible mushrooms and symbiotic growth-promoting microorganisms was revealed.

From the results of this experiment, there was a significant effect in promoting the growth of *H. marmoreus* mycelium on PDA plates with the addition of 5% sterile fermentation filtrate (Figure 1 and Figure S1). Such growth-promoting effects may be common in nature. They are not limited to microorganisms. Wang et al. also found that a strain of *B. subtilis*, WXCDD105, stimulated both tomato seed germination and seedling growth while having strong antagonistic effects on *Botrytis cinerea* [25]. Kai et al. demonstrated that the promoting effect of *S. odorifera* rhizobacteria volatiles on the moss *Physcomitrella patens* was attributed to CO_2_ as the dominant component of the volatile mixture [26]. However, whether the carbon cycle of *S. odorifera* had an effect on the growth of *H. marmoreus* through its sterile fermentation filtrate in this experiment is still under investigation. The validation of the back supplementation of the main substances in the fermentation filtrate showed that the aseptic fermentation filtrate HZSO-1 had the best probiotic effect compared to the single-standard and mixed-standard treatments (Figure 1), which also seemed to be an irreplaceable aspect of chemically synthesized probiotic substances. The clamp junction is a special way of mycelial union in the mycelial differentiation stage, through which the two-cell cells divide continuously, and thus, the tip of the mycelium extends anteriorly. In *Pleurotus eryngii*, overexpression of *GNAI* increases the number of clamp junctions of the mycelium and promotes mycelial growth and primordium formation [21]. In this experiment, a significant increase in the number of clamp junctions was found after HZSO-1 treatment (*p* < 0.05) (Figure 2). This provided the conditions for further mycelial bending to form the primordium. This also further explains that adding sterile fermentation filtrate from *S. odorifera* promoted the fruiting body growth of *H. marmoreus* [6]. In addition, Kim et al. found that *Pseudomonas* sp. strain P7014 and its supernatant promoted the faster growth of *Pleurotus eryngii* mycelium and induced the primordial formation one day earlier [27].

Metabolomics studies are concerned with the metabolome, the collection of all small molecule metabolites in a biological sample. The metabolome is at the most downstream stage of the activity of life, and the majority of small molecule metabolites are the result of multi-step enzymatic reactions [28,29]. Therefore, in addition to genetic factors, any environmental perturbation and spatiotemporal changes can alter the metabolome, while these metabolite changes, in turn, affect organismal gene expression, growth differentiation, function, and behavior [30,31]. Metabolomics has been used less to study interactions between bacteria and edible mushrooms than to study small molecule metabolites in humans and common medicinal plants. The secondary metabolites of symbiotic bacteria are much more diverse and complex and varied than those of animals and common plants because symbiotic bacteria can adapt their metabolism to a variety of environments and engage in interspecific competition and exchange [29,31,32]. In this experiment, UHPLC-QE-MS, a non-target metabonomics analysis method, was used to investigate the effects of sterile fermentation filtrate of *S. odorifera* and QS signal molecular standard treatment on the metabolomics of *H. marmoreus*. By means of KEGG enrichment, we found a significant upregulation of the pentose phosphate pathway in the carbohydrate metabolism in the HZSO-1, cyclo(Pro-Leu), 3-oxo-C_6_-HSL, and mix treatment groups (Figure 5). The metabolism of carbon sources is the most important for nutrient degradation and absorption in edible mushrooms. Carbon metabolism not only provides raw materials for the biosynthesis of carbohydrates and amino acids in edible mushrooms but is also the main source of energy for the maintenance of the growth of edible mushrooms [33]. In the cultivation of edible mushrooms, agricultural wastes such as wood chips, cottonseed hulls, rice hulls, and bagasse have often been used as the main material. These materials contain a large amount of cellulose and hemicellulose. Edible mushrooms rely on extracellular enzymes such as cellulase and xylanase to degrade cellulose and hemicellulose to produce monosaccharides that enter the central carbon metabolism (EMP, PPP, and TCA cycle) to provide energy and materials for mycelial growth and development [34,35]. In a recent study, QS autoinducers were found to increase the transcript level of lignin-degrading enzyme genes in *H. marmoreus* [20]. It is further speculated that the addition of sterile fermentation filtrate of *S. odorifera* may promote the mycelial growth of *H. marmoreus* by regulating the carbon metabolism cycle. A previous study of ours also showed that intracellular and extracellular enzymes of carbon metabolism in edible mushrooms are closely related to mycelial growth rate and mushroom yield [36]. As shown in Figures S6 and S7, ribose 5-P was upregulated, which promoted the PPP pathway and increased the synthesis of fructose 6-P, which is an important link between the PPP pathway and the EMP pathway. This would promote the EMP pathway, but the two downstream substances, 2-phosphoglycerate and phosphoenolpyruvate (PEP), were significantly downregulated (*p* < 0.05) (Figures S6 and S7). It was hypothesized that the metabolism of PEP would be faster than its synthesis, which would also contribute to the efficiency of the downstream TCA cycle in mitochondria. This could generate a large amount of energy to promote mycelial growth and fruiting body development. Judging from the significant upregulation of the ATP/ADP ratio (*p* < 0.05) (Figures S6 and S7), HZSO-1 treatment could promote carbon metabolism and provide energy for mycelial growth and development of *H. marmoreus* through the above-mentioned pathway. This idea is further supported by the fact that 2-oxoglutarate was upregulated in the TCA cycle (Figures S6 and S7) and could bridge carbon and amino acid metabolism. Meanwhile, the downregulation of glutamate and upregulation of ornithine in the upstream (Figures S6 and S7) also seemed to promote the flow of the TCA cycle. Lin et al. also found the same result: low PPP metabolism level and unbalanced distribution of CCM in *H. marmoreus* were the two main factors contributing to the slow assimilation of carbon source and low mycelial growth rate [37]. It has been reported that QS signaling molecules have important effects on the physiological metabolism of microorganisms. N-acyl homoserine lactones (AHLs), as a typical representative, play an important role in the regulation of substance and energy metabolism of bacteria [14]. Therefore, we speculated that QS signaling molecules also have an effect on the physiological metabolism of *H. marmoreus*. There was a higher concentration of DAMs in the amino acid pathway after HZSO-1 treatment compared to the control (Figure 5). This provides further evidence that HZSO-1 treatment affected the amino acid pathway of the mycelium. In the five comparison groups, metabolic pathways and cofactor biosynthesis were common metabolic pathways with significant differences. The changes in metabolite expression in the above common pathways may reveal the influence of QS signaling molecules on mycelial growth. Moreover, the ATP-binding cassette (ABC) transporter protein was the most significant difference from the control group after HZSO-1 treatment (Figure 5). ABC transporters are ubiquitous proteins in fungi [38], and they could use ATP energy for substrate transfer across biological membranes [39]. It has been suggested that quorum sensing is mediated by small signaling molecules that accumulate in the extracellular environment. The signal transduction mechanism of accumulation of these molecules in the medium is through passive diffusion across the membrane, involving efflux pumps and specific transporters [40]. We hypothesized that the ABC transporters would play a similar role in the response of the *H. marmoreus* mycelium to HZSO-1.

## 5. Conclusions

In this paper, we investigated the pro-growth mechanism of *H. marmoreus* by exogenously adding sterile fermentation filtrate of *S. odorifera* and QS signaling molecules. The addition of exogenous substances could significantly promote mycelial growth, increase the number of clamp junctions, and also make the mycelium more robust (*p* < 0.05). Metabolomic analysis revealed that the differential metabolites after the above treatments were mainly enriched in carbohydrate and amino acid metabolism pathways. Specifically, mycelial growth and fruiting body development could activate the PPP pathway, thereby increasing the efficiency of the TCA cycle to obtain more energy from HZSO-1 treatment. However, the balance between the PPP, EMP, and TCA cycles in the central carbon metabolism still needs to be further investigated. This study may provide a basis for further elucidating the mechanism of symbiotic bacterial effects on *H. marmoreus* growth and development.

## Data Availability

The raw/processed data required to reproduce these findings cannot be shared at this time as the data also form part of an ongoing study.

## Supplementary Materials

The following supporting information can be downloaded at https://www.mdpi.com/article/10.3390/biom13121804/s1, Figure S1: Effects of sterile fermentation broth of *S. odorifera* on the growth of *H. marmoreus* hyphae on the plate. (**A**) Diagram and (**B**) rate of growth of *H. marmoreus* hyphae (mm/d) after treatment with the sterile fermentation broth at different concentrations. Figure S2: Effects of different signaling molecules on plate growth of *H. marmoreus* hyphae. (**A**) Diagram and (**B**) mycelial growth rate (mm/d) of different signaling molecule treatments on *H. marmoreus* growth. Figure S3: Effect of mixture (3-oxo-C_6_-HSL: cyclo(Pro-Leu):cyclo(Tyr-Leu) in 1:1:1 ratio) on plate growth of *H. marmoreus* hyphae. (**A**) Graph and (**B**) mycelial growth rate (mm/d) at different concentrations. Figure S4: Total ion chromatograph of the QC sample. (**A**) Positive ion mode and (**B**) negative ion mode. Figure S5: Multivariate statistical analysis of *H. marmoreus* mycelia. (**A**) PCA in positive ion mode; (**B**) PCA in negative ion mode; (**C**) PLS-DA in positive ion mode; (**D**) PLS-DA in negative ion mode. Figure S6: Heat maps of differential accumulation of metabolites in *H. marmoreus* mycelium samples in five groups. (**A**) HZSO-1 vs. control; (**B**) cyclo(Pro-Leu) vs. control; (**C**) cy-clo(Tyr-Leu) vs. control; (**D**) 3-oxo-C6-HSL vs. control; (**E**) mix vs. control. Figure S7: Schematic diagram of the metabolic pathways of carbohydrate and amino acid metabolism in *H. marmoreus* as influenced by the sterile fermentation filtrate of *S. odorifera*.
