# Supplementary material for: The Promoting Mechanism of the Sterile Fermentation Filtrate of Serratia odorifera on Hypsizygus marmoreus by Means of Metabolomics Analysis
Source: Biomolecules. 2023 Dec 18;13(12):1804. doi: 10.3390/biom13121804 (PMC10741993; doi:10.3390/biom13121804)
Supplement: Supplementary file 1 [file biomolecules-13-01804-s001.zip › biomolecules-2758446-supplementary.pdf]

Supplementary Information

The promoting mechanism of the sterile fermentation filtrate of *Serratia odorifera* on *Hypsizygus marmoreus* by means of metabolomics analysis

Jixuan Cao [1], Jiacheng Xie [1], Mingming Yu [1], Tao Xu [1], Huangru Zhang [1], Liding Chen [1] and Shujing Sun [1, 2,*]

[1] College of Life Sciences, Fujian Agriculture and Forestry University, Fuzhou 350002 , China; 2210514023@fafu.edu.cn (J.C.); 3200537080@fafu.edu.cn (J.X.); 1210514112@fafu.edu.cn (M.Y.); 3215413137@stu.fafu.edu.cn(T.X.);3205403014@stu.fafu.edu.cn (H.Z.); chenliding@fafu.edu.cn (L.C.)

[2] Gutian Edible Fungi Research Institute, Fujian Agriculture and Forestry University, Ningde 352200, China

* Correspondence: shjsun2004@126.com

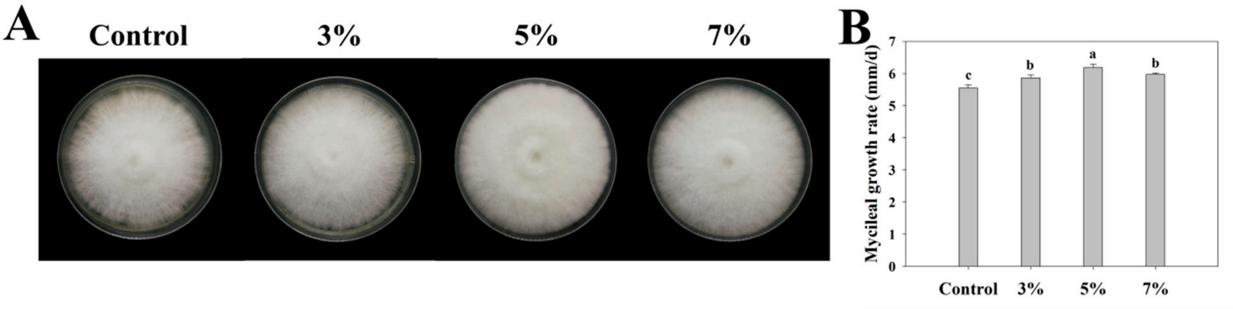

Figure S1 Effects of sterile fermentation broth of *S. odorifera* on the growth of *H. marmoreus* hyphae on the plate. (A) Diagram and (B) rate of growth of *H. marmoreus* hyphae (mm/d) after treatment with the sterile fermentation broth at different concentrations.

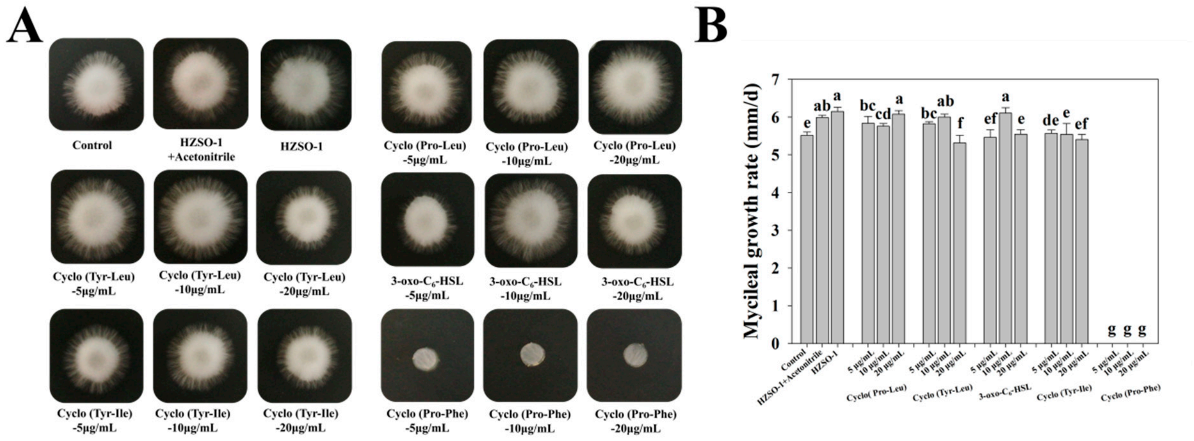

Figure S2 Effects of different signaling molecules on plate growth of *H. marmoreus* hyphae. (A) Diagram and (B) mycelial growth rate (mm/d) of different signaling molecule treatments on *H. marmoreus* growth.

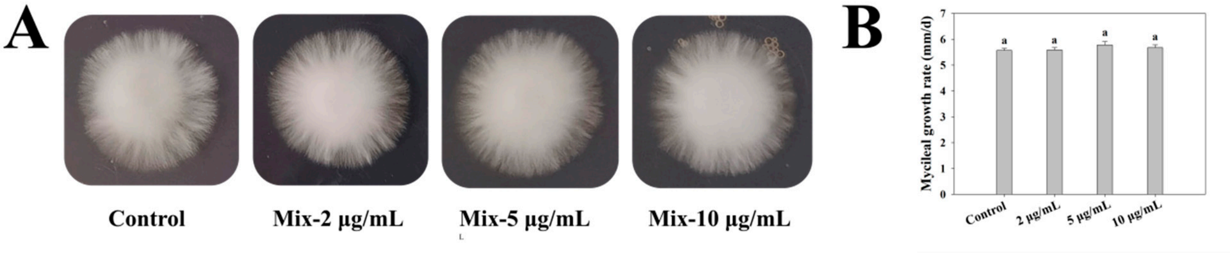

Figure S3 Effect of mixture (3-oxo-C_6-HSL: cyclo(Pro-Leu): cyclo(Tyr-Leu) in 1:1:1 ratio) on plate growth of *H. marmoreus* hyphae. (A) Graph and (B) mycelial growth rate (mm/d) at different concentrations.

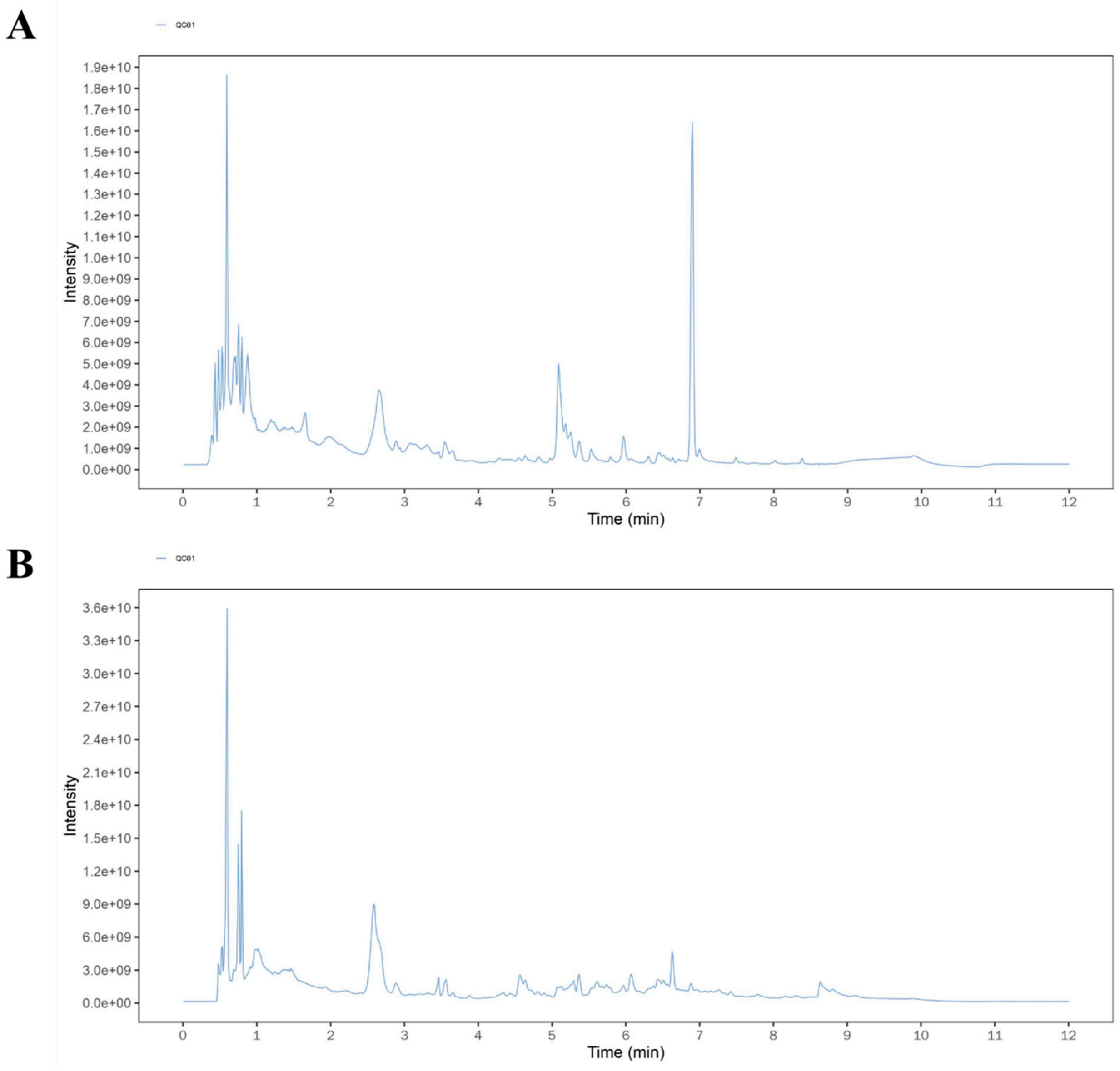

Figure S4 Total ion chromatograph of the QC sample. (A) Positive ion mode and (B) negative ion mode.

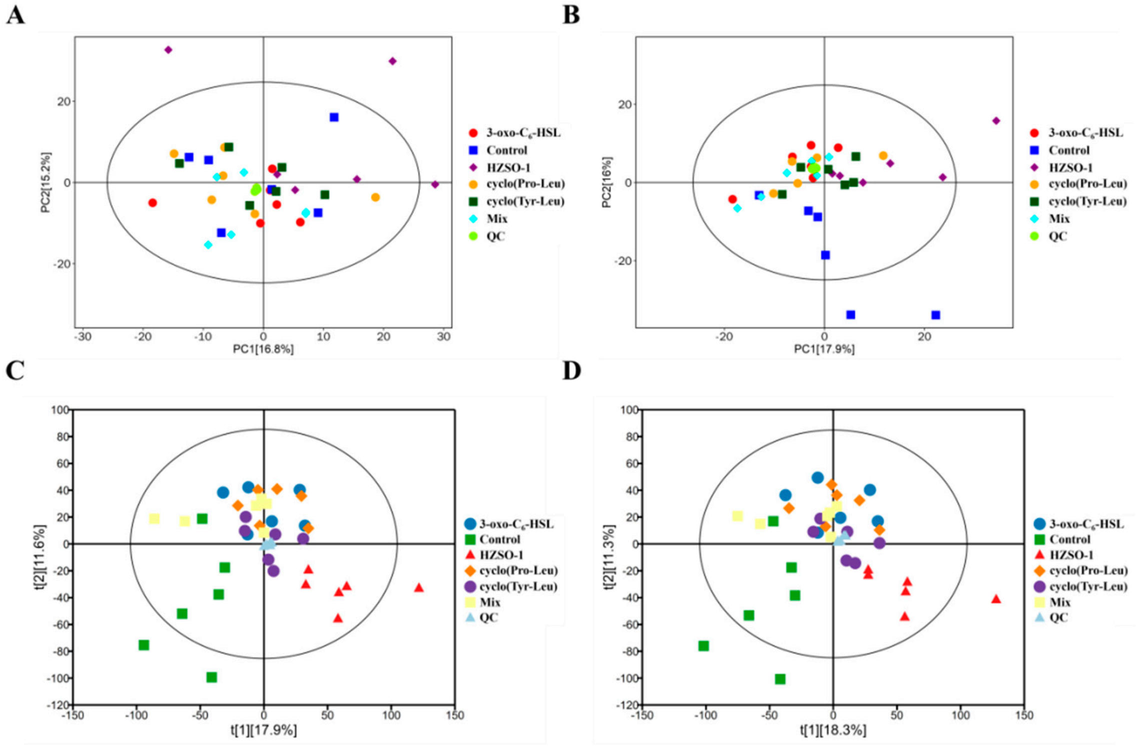

Figure S5 Multivariate statistical analysis of *H. marmoreus* mycelia. (A) PCA in positive ion mode; (B) PCA in negative ion mode; (C) PLS-DA in positive ion mode; (D) PLS-DA in negative ion mode.

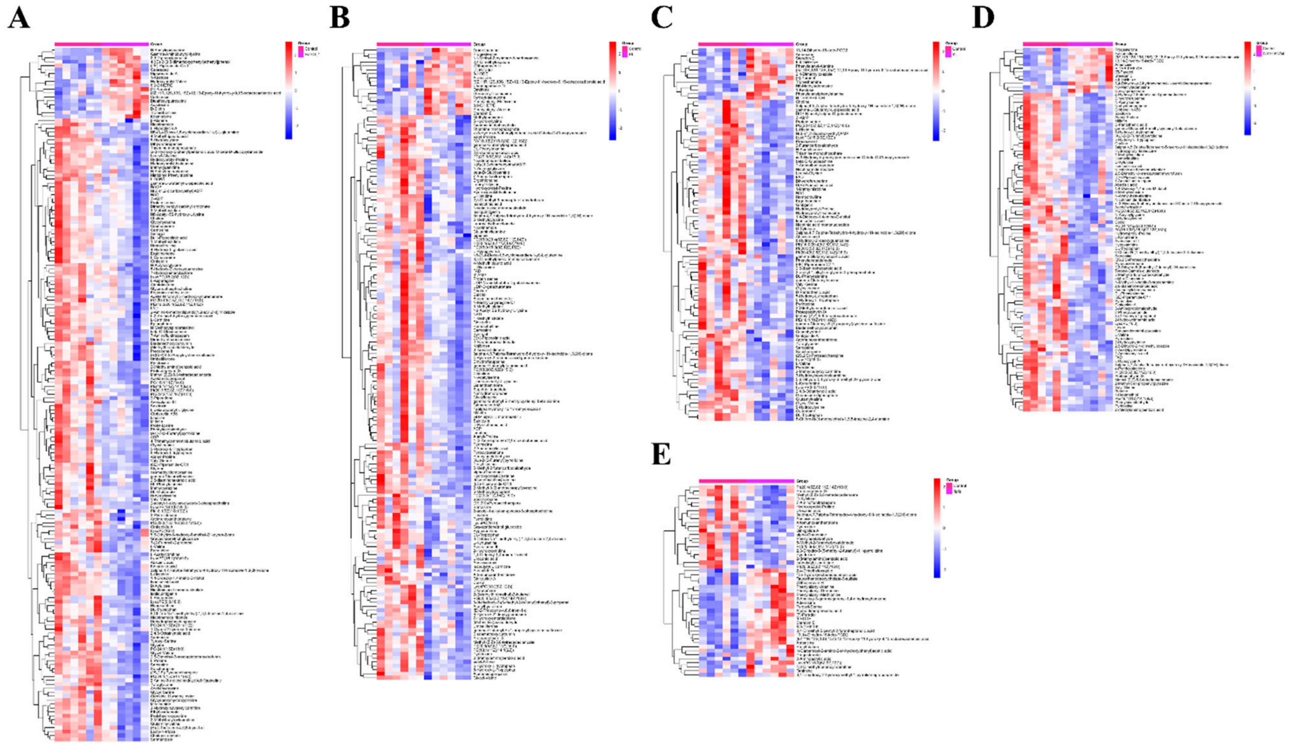

Figure S6 Heat maps of differential accumulation of metabolites in H. marmoreus mycelium sam-ples in five groups. (A) HZSO-1 vs. control; (B) cyclo(Pro-Leu) vs. control; (C) cy-clo(Tyr-Leu) vs. control; (D) 3-oxo-C6-HSL vs. control; (E) mix vs. control.

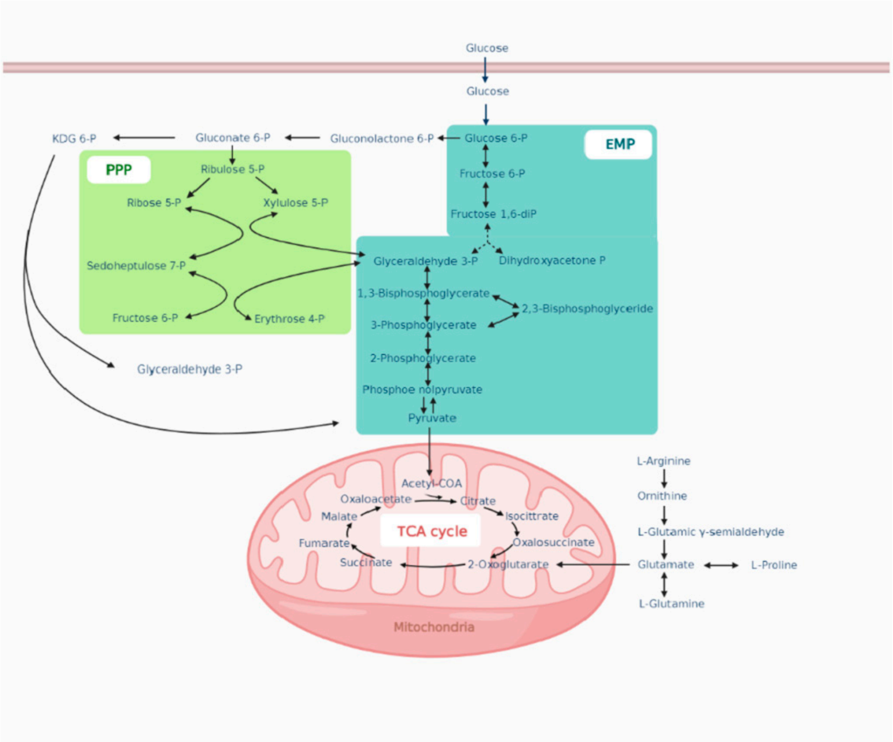

Figure S7 Schematic diagram of the metabolic pathways of carbohydrate and amino acid metabolism in *H. marmoreus* as influenced by the sterile fermentation filtrate of *S. odorifera*.
